# Neutrophil but not lymphocyte response to matched interval and continuous running differs between protocols and sex

**DOI:** 10.1007/s00421-024-05675-0

**Published:** 2024-12-03

**Authors:** F. Adammek, S. Belen, A. Metcalfe, F. Weißhaar, N. Joisten, D. Walzik, P. Zimmer

**Affiliations:** 1 Division of Performance and Health (Sports Medicine), Institute for Sport and Sport Science, Technical University Dortmund, Otto-Hahn-Straße 3, 44227 Dortmund, Germany; 2 Institute of Cardiovascular Research and Sport Medicine, German Sport University Cologne, Cologne, Germany

**Keywords:** Circulating immune cells, Interval running, Continuous running, Matched exercise, Sex differences, Cortisol

## Abstract

When considering the acute effects of different modalities of endurance training on cellular immune components, the matching of duration and mean intensity and the consideration of sex-dependent differences have received less attention so far. The aim of the study was to compare the effects of interval running (IR) vs. continuous running (CR) on circulating immune cells and cellular immune inflammation markers. Additionally, we analyze sex-dependent differences in these effects. In a cross-over study design, 24 recreational runners completed a mean intensity and duration-matched session of IR and CR. Blood samples were taken immediately before, after, and 1 h after exercise cessation for cell counts and cortisol level quantification. Neutrophil counts showed a sustained increase after both exercise modalities with significantly greater values after IR compared to CR at 1 h after exercise and greater increases in females. Neutrophil-to-lymphocyte ratio and systemic immune-inflammation index increased 1 h after exercise with significantly higher values after IR compared to CR with greater values in females. Platelet-to-lymphocyte ratio decreased in both immediately after IR and 1 h after exercise in both sexes and modalities. The cortisol level shows a lower value immediately after CR compared to IR with no sex-dependent differences. The IR protocol induces greater immune cell mobilizations in comparison to CR, which is primarily based on a systemic increase in neutrophil counts. Neutrophil-to-lymphocyte ratio and systemic immune-inflammation index are suitable to distinguish this effect between training modalities. Sex-dependent differences confirm that biological sex influences the immune cells response to acute exercise.

**Protocol registration: **German Clinical Trials Register, DRKS00017686, Date of registration: 11.02.2020.

**Supplementary Information:**

The online version contains supplementary material available at 10.1007/s00421-024-05675-0.

## Introduction

Acute bouts of physical exercise induce strong transient alterations in circulating leukocytes (LEUK) and stress hormones (e.g. cortisol) (Fragala et al. 2011). These alterations affect the amount and function of circulating immune cells and are dependent mainly on the intensity and duration of exercise (Gleeson et al. 2011). The inflammatory states induced by such exercise responses are highly relevant since they are associated with both physiological conditions and adaptations, such as tissue repair, as well as with the onset of pathophysiological conditions and overtraining (Gleeson et al. 2011; Walzik et al. 2021; Golia et al. 2014). To generally reflect cellular inflammatory states in response to acute exercise, the use of the integrative markers neutrophil-to-lymphocyte ratio (NLR = neutrophils:lymphocytes), platelet-to-lymphocyte ratio (PLR = platelets:lymphocytes) and the systemic immune-inflammation-index (SII = neutrophils:lymphocytes × platelets) has proven effective, while no additional infrastructural and financial resources and only low temporal expenditure are required for assessment and calculation (Walzik et al. 2021). A deeper understanding of the acute effects of exercise on cellular immune components, integrative markers, and stress hormones such as cortisol may improve exercise load management and recovery strategies by enhancing adaptation, optimizing athletic performance, and promoting long-term health.

An important aspect in this context is the training modality, since stronger perturbations are associated with higher intensities and longer durations (Walsh et al. 2011a, 2011b). Investigating frequently used endurance training protocols such as interval and continuous training is challenging in regards of their comparability. Namely, most studies did not accurately match intensity and duration. The complexity arises from the more intricate structure of interval exercise compared to continuous ones, as there will always be a matching discrepancy between interval and continuous exercises regarding to duration, peak or mean intensity (Hofmann and Tschakert 2017).

Besides exercise modality related variables (e.g. intensity and duration), earlier literature suggests that biological sex may also affects the immune cell response of single immune components, mainly lymphocytes (LYM), to acute exercise, with stronger effects in females (Timmons et al. 2005; Moyna et al. 1996; Fragala et al. 2011). Compared to males, females reveal an enhanced innate and acquired immune response to infectious diseases and vaccines (Pennell et al. 2012; Klein and Flanagan 2016). However, sex-dependent differences in exercise-induced alteration have been investigated much less extensively, particularly when comparing exercise modalities.

To date, only Bogdanis et al. (2022) have conducted a study matching mean intensity and duration, incorporating a relatively low mean intensity [49% of maximal oxygen uptake (${\dot{\text{V}}}$O_2_max)], but neglecting sex-dependent differences. Their results showed that only intervals of sufficient length (60 s) in the peak intensity phase provide stronger immune cell perturbations compared to shorter intervals (10 s, 30 s) or continuous endurance exercise (Bogdanis et al. 2022). Classical high-intensity interval exercise programs, which usually demand a higher mean intensity (around 70% ${\dot{\text{V}}}$˙O_2_max) have not been investigated yet. It is noteworthy that further literature dealing with protocol specific cellular immune responses neglects sex-dependent differences (Arroyo et al. 2022; Elmer et al. 2016; Jamurtas et al. 2018; Da Neves et al. 2015; Bogdanis et al. 2022).

To examine exercise effects on cellular immune responses based on frequently used endurance exercise protocols, this explorative cross-over study compared a single bout of interval running (IR) versus continuous running (CR), matched for a duration of 50 min at a mean intensity of 70% ${\dot{\text{V}}}$˙O_2_max. Additionally, we investigate sex-dependent differences of exercise-induced effects in circulating immune cells and their corresponding ratios (NLR, PLR, SII).

We hypothesize that IR will have a greater impact on the response of circulating immune cells compared to duration and mean intensity matched CR. Furthermore, we expect greater immune cell perturbations after exercise in females than males independent of the running protocol.

## Methods

**Fig. 1:**
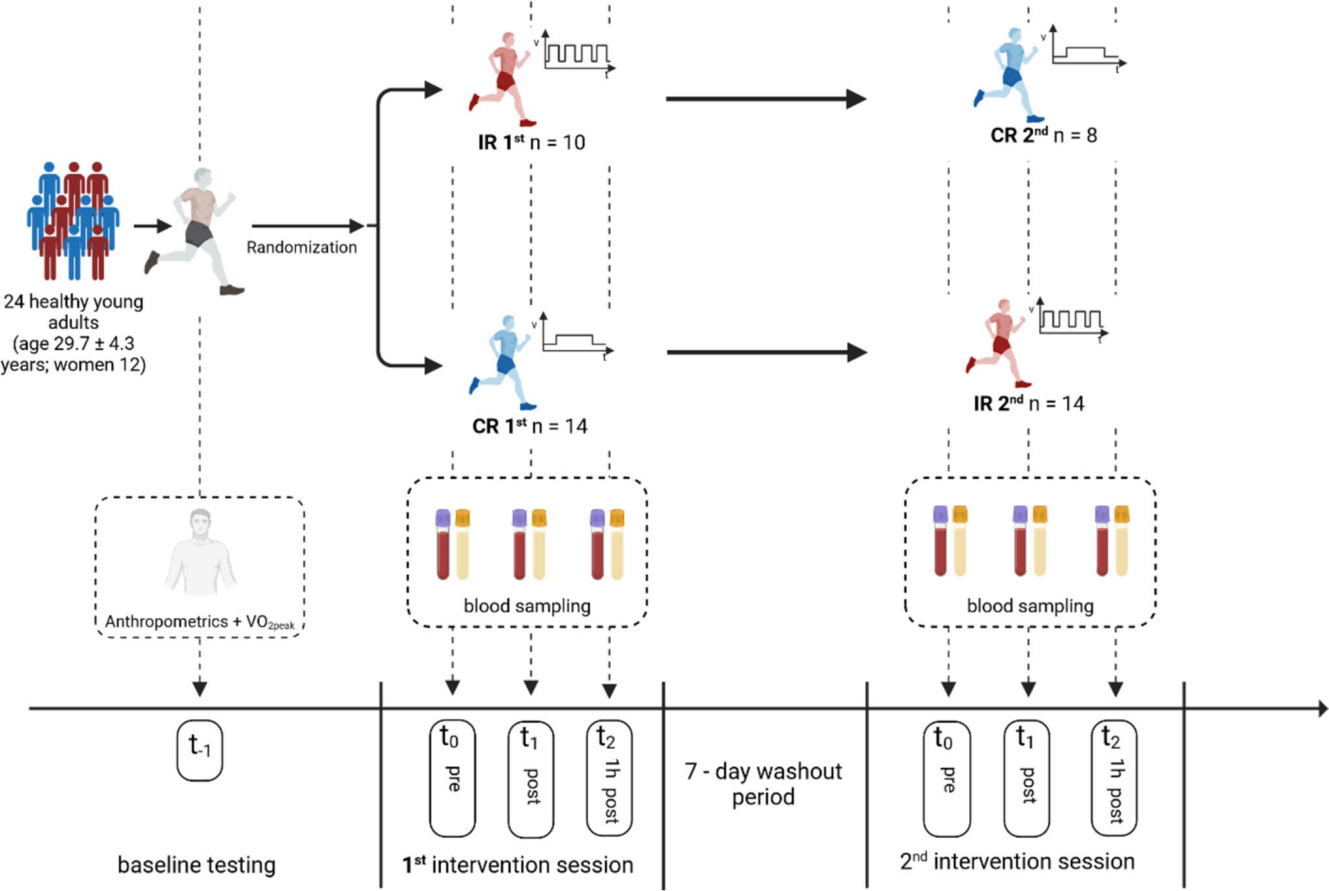
Schematic overview of the study design

To investigate this hypothesis, a serial randomized crossover study was conducted (Fig. 1). The study was approved by the local ethics committee of the German Sport University Cologne and registered under DRKS00017686. All subjects signed a written informed consent prior to participation. In total, 24 recreational runners (12 female and 12 male) were included. The inclusion and exclusion criteria are shown in Table 1.

**Table 1:** Inclusion and exclusion criteria of the study

Inclusion criteria	Exclusion criteria
• Body mass index < 30 • Recreationally active runners (running between 2 and 5 h per week) • 18–35 years	• Muscle disorder • Cardiac or kidney disease • Taking medication (e.g. anti-inflammatory drugs, antibiotics) or nutritional supplements

For baseline exercise testing, all participants completed an incremental running test on a motorized treadmill (Woodway PPS Med, Germany) until exhaustion to assess peak oxygen uptake (${\dot{\text{V}}}$O_2_peak), which was used to match the exercise intensities (respective running speed) for the exercise sessions. The test protocol consisted of a 5-min warm-up period at ~6–8 km/h, followed by increments of 1 km/h every minute until participants were not able to maintain the speed. To verify measured ${\dot{\text{V}}}$O_2_peak values, an additional verification test until exhaustion was performed five minutes after cessation. The verification consisted of running until exhaustion at one stage higher (X + 1 km/h) than the last completed one during the incremental test. Both tests began after warm-up period at 8 km/h before increasing the speed as mentioned above.

Participants were randomized to either Sequence 1 (IR-CR) or Sequence 2 (CR-IR) via a minimization procedure (Scott et al. 2002) using the software ‚Randomization-In-Treatment-Arms’ (RITA, Version 1.51, Germany) with BMI, ${\dot{\text{V}}}$˙O_2_peak and age as stratification factors.

At least 3 days after baseline testing, the participants conducted the first exercise condition. After a washout period of 7 days, subjects visited the laboratory again to complete the other exercise condition. IR condition consisted of six interval bouts with three minutes at a running speed corresponding to 90% ${\dot{\text{V}}}$O_2_peak, alternated with three minutes bouts at a running speed corresponding to 50% of ${\dot{\text{V}}}$˙O_2_peak. The session started and finished with a seven minute warm-up and cool-down at a running speed corresponding to 70% ${\dot{\text{V}}}$˙O_2_peak. Including warm-up and cool-down, the average exercise session intensity resulted in 70% of ${\dot{\text{V}}}$˙O_2_peak for the total session duration of 50 min. The CR condition consisted of 50 min running at a speed corresponding to 70% ${\dot{\text{V}}}$˙O_2_peak. In this way, IR and CR were matched for duration (50 min), mean intensity (70% ${\dot{\text{V}}}$O_2_peak), and total running distance (9843 ± 494 m) (Bartlett et al. 2012).

For all visits, participants were asked to refrain from caffeine, alcohol, and exercise 24 h before testing as well as arrive on an empty stomach (no food intake for at least 2 h). All exercise sessions were accomplished at the same daytime, and on the same treadmill, which was set at a 1% incline (Jones and Doust 1996).

Venous blood samples were collected before (pre, T_0_), immediately after (post, T_1_), and 1 h after each exercise session (1 h post, T_2_). Blood cell count analysis of LEUK, LYM, neutrophils (NEUT), platelets (PLT) was measured from EDTA blood using a hematology analyzer (Sysmex KX-21N). Blood cell counts were used to calculate NLR (NEUT [10^9^/L] × LYM [10^9^/L]^−1^), PLR (PLT [10^9^/L] × LYM [10^9^/L]^−1^), and SII (NLR × PLT [10^9^/L]).

To separate serum from whole blood, samples were centrifuged at 3500 rpm for 15 min and were stored at −80 ℃ until analysis. Serum concentrations of Cortisol were quantified using Cortisol immunoassay (R&D Systems, USA) according to the manufacturer`s instructions.

To determine main effects for time as well as time × condition interaction effects (3 × 2 design) on circulating immune cells and their corresponding ratios mixed effect analyses of variances (mixed-effect ANOVAs) were conducted accounting for intraindividual variances. Contrasts were set to determine the differences between the periods T_0_ – T_1_ (exercise) and T_1_ – T_2_ (recovery), and to assess whether these effects differed between exercise conditions. For statistically significant contrasts, paired, respectively, unpaired *t*-test were used to statistically test for differences between T_0-_T_2_ and between conditions. To further investigate differences of groups by sex over time (3 × 4 design), we performed a baseline adjusted mixed-effect analysis of covariances (mixed-effect ANCOVAs) accounting for sex differences at baseline and intraindividual variances. Contrasts were used to determine differences between the periods T_0_ – T_1_ (exercise) and T_1_ – T_2_ (recovery), and to assess whether these effects differed between sexes within the same condition (e.g. females IR—males IR). For statistically significant contrasts, paired, respectively, unpaired *t*-tests were used to statistically test for differences between T_0_ – T_2_ and between groups.

All statistical analyses were corrected for multiple comparisons using the false discovery rate (FDR) correction (Benjamini and Hochberg 1995). The level of significance was set at *p* < 0.05 for all analyses. Data were log10 transformed and normal distribution as well as homogeneity of variances was evaluated using the Shapiro–Wilk, respectively, the Levene test. In case of violation, robust ANOVAS and ANCOVAS were conducted. For robust *t*-tests, robust standard errors (SE) with z-ratio was employed to compare the means. Sphericity assumptions were checked using the Mauchly test and results were corrected according to Greenhouse–Geisser if violated. Missing data points were deleted listwise prior to analysis. Statistical analyses were conducted using the rstatix (Kassambara 2021), lme4 (Bates et al. 2015), robustlmm (Koller 2016), parameters (Lüdecke et al. 2022) and emmeans (Russell et al. 2023) package in R Studio Version 2022.7.2.576 (RStudio Team 2019).

## Results

Participant characteristics (*n* = 24) are listed in Table 2. One blood draw failed at 1 h post exercise in one participant after the CR condition. Tables of mean values and standard deviation of variables by condition (Online Resource 1) and by condition and sex (Online Resource 2) are available as supplementary material.

**Table 2:** Participant characteristics and verification test

Parameter	Overall (*n* = 24)	Male (*n* = 12)	Female (*n* = 12)
	mean ± SD	mean ± SD	mean ± SD
Age (years)	29.67 ± 4.28	31.58 ± 3.72	27.75 ± 4.00
Weight (kg)	69.70 ± 11.05	77.50 ± 8.77	61.91 ± 6.78
BMI (kg/m^2^)	22.20 ± 2.36	23.36 ± 1.90	21.05 ± 2.23
${\dot{\text{V}}}$O_2_peak (ml·min^−1^·kg^−1^)	56.64 ± 6.36	58.86 ± 7.48	54.42 ± 4.04
${\dot{\text{V}}}$˙O_2_peak B (ml·min^−1^·kg^−1^)	53.71 ± 8.35	57.05 ± 7.58	50.38 ± 7.86
TTE (s)	80.42 ± 16.70	81.17 ± 18.90	79.67 ± 14.54
HR_max_ (bpm)	181.44 ± 11.14	179.58 ± 11.21	183.46 ± 10.97
RPE_max_	19.38 ± 1.00	19.42 ± 1.14	19.33 ± 0.87

Data are presented by means and standard deviation (SD)

*BMI* body-mass-index, ${\dot{{V}}}$*O*_*2*peak_* B* verification result of ${\dot{\text{V}}}$˙O_2_peak test, *TTE* time to exhaustion in verification test, *HR*_*max*_ maximum heart rate in ${\dot{\text{V}}}$O_2_peak test, *RPE*_*max*_ maximum rate of perceived exertion in ${\dot{\text{V}}}$O_2_peak test

**Fig. 2:**
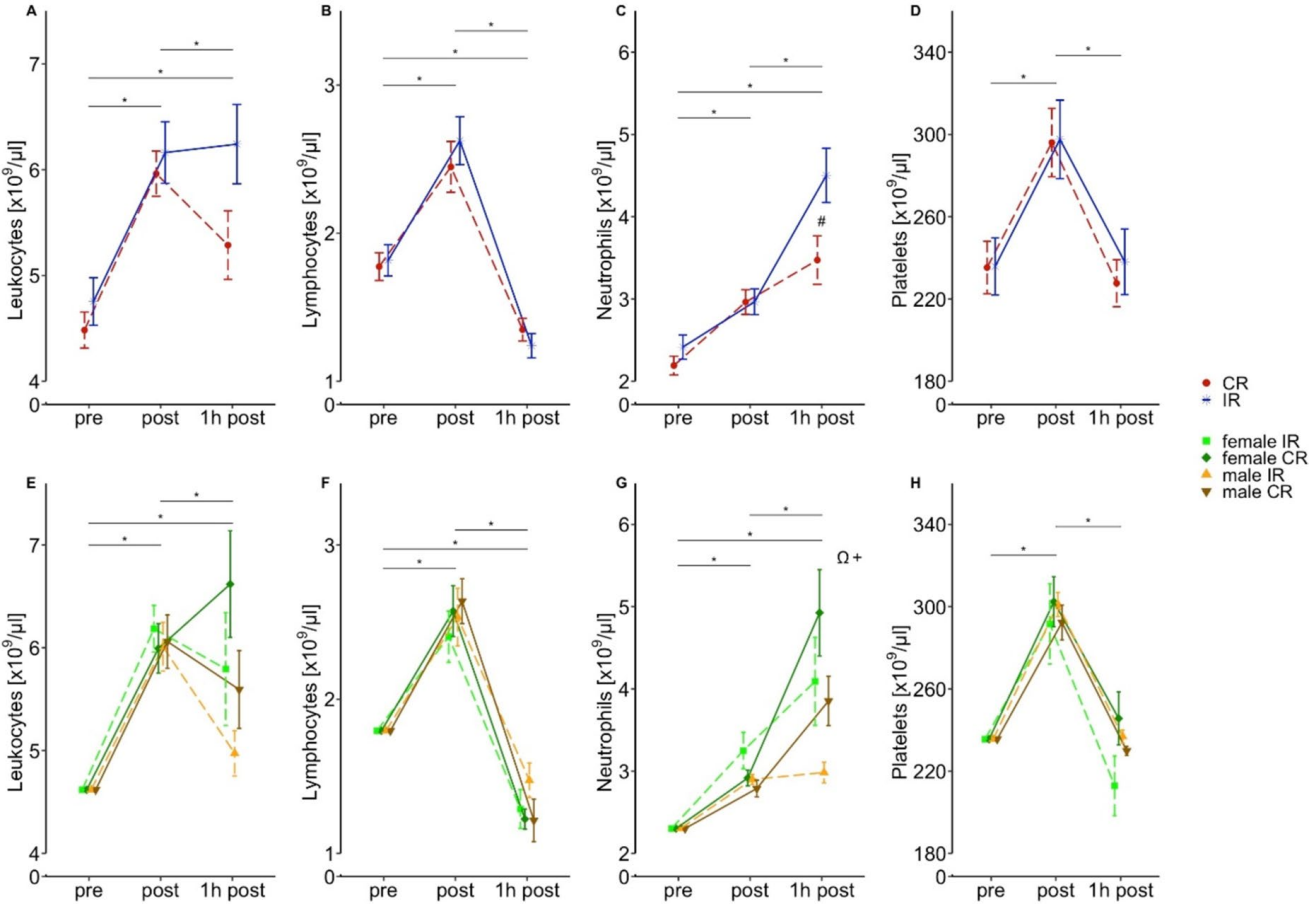
Effects of exercise conditions and sex-dependent differences on immune cell populations. Data are presented as means ± SE. * indicates significant main effects of time (*p* < 0.05). **#** indicates significant time × condition interaction effects (*p* < 0.05). Significance codes for sex-dependent interactions (*p* < 0.05): Ω = female IR vs. male IR, + = female CR vs. male CR

In LEUK counts exercise induced a significant increase immediately post-exercise cessation, without differences between conditions which overall decreased slightly 1 h post exercise, while remaining higher compared to baseline (Fig. 2a; Tables 3, 4). However, there was no difference between conditions (Fig. 2a; Table 3). Regarding sex-dependent differences after exercise, no statistically significant difference was observed between males and females performing IR or CR (Fig. 2e; Table 3).

**Table 3:** Linear mixed effects model analyses

Fixed effects					Random effects		Baseline adjusted fixed effects					Random effects	
Contrast	Coefficient	se	Statistic	*p*	Parameter	sd	Contrast	Coefficient	se	Statistic	*p*	Parameter	sd
Leukocytes							*Leukocytes*						
(Intercept)	0.718	0.016	46.332	<0.001*	Participant	0.068	(Intercept)	0.173	0.046	3.779	0.001*	Participant	0.018
Exercise (T_0_ − T_1_)	0.119	0.015	7.718	<0.001*	Residual	0.075	Baseline	0.827	0.069	11.968	<0.001*	Residual	0.061
Recovery (T_1_ – T_2_)	−0.048	0.023	−2.080	0.059			Exercise (T_0_ − T_1_)	0.117	0.013	9.121	<0.001*		
Exercise condition × exercise	0.007	0.015	0.455	0.650			Recovery (T_1_ – T_2_)	−0.055	0.019	−2.837	0.011*		
Exercise condition × recovery	−0.045	0.023	−1.954	0.063			Female IR-male IR × exercise	−0.002	0.036	−0.058	0.954		
							Female CR-male CR × exercise	−0.015	0.036	−0.399	0.767		
							Female IR-male IR × recovery	−0.086	0.055	−1.582	0.227		
							Female CR-male CR × recovery	−0.043	0.055	−0.771	0.551		
Lymphocytes							*Lymphocytes*						
(Intercept)	0.191	0.022	8.815	<0.001*	Participant	0.100	(Intercept)	−0.020	0.014	−1.467	0.356	Participant	0.000
Exercise (T_0_ − T_1_)	0.144	0.016	8.925	<0.001*	Residual	0.079	Baseline	0.890	0.052	17.260	<0.001*	Residual	0.071
Recovery (T_1_ – T_2_)	−0.430	0.024	−17.595	<0.001*			Exercise (T_0_ − T_1_)	0.144	0.015	9.727	<0.001*		
Exercise condition × exercise	−0.012	0.016	−0.728	0.468			Recovery (T_1_ – T_2_)	−0.429	0.022	−19.133	<0.001*		
Exercise condition × recovery	0.059	0.024	2.432	0.025*			Female IR-male IR × exercise	0.008	0.042	0.183	0.998		
							Female CR-male CR × exercise	0.000	0.042	0.003	0.998		
							Female IR-male IR × recovery	−0.001	0.063	−0.020	0.998		
							Female CR-male CR × recovery	0.058	0.064	0.914	0.691		
Neutrophils							*Neutrophils*						
(Intercept)	0.476	0.018	26.066	<0.001*	Participant	0.076	(Intercept)	0.190	0.018	10.333	<0.001*	Participant	0.000
Exercise (T_0_ − T_1_)	0.112	0.022	5.220	<0.001*	Residual	0.105	Baseline	0.826	0.050	16.508	<0.001*	Residual	0.065
Recovery (T_1_ – T_2_)	0.165	0.032	5.068	<0.001*			Exercise (T_0_ − T_1_)	0.106	0.014	7.800	<0.001*		
Exercise condition × exercise	0.020	0.022	0.950	0.344			Recovery (T_1_ – T_2_)	0.179	0.020	8.739	<0.001*		
Exercise condition × recovery	−0.089	0.032	−2.726	0.009*			Female IR-male IR × exercise	−0.035	0.038	−0.924	0.395		
							Female CR-male CR × exercise	−0.032	0.038	−0.829	0.407		
							Female IR-male IR × recovery	−0.135	0.057	−2.352	0.023*		
							Female CR-male CR × recovery	−0.221	0.058	−3.811	<0.001*		
Platelets							*Platelets*						
(Intercept)	2.368	0.024	99.448	<0.001*	Participant	0.115	(Intercept)	0.011	0.039	0.280	0.866	Participant	0.000
Exercise (T_0_ − T_1_)	0.099	0.009	10.629	<0.001*	Residual	0.046	Baseline	1.001	0.016	61.340	<0.001*	Residual	0.021
Recovery (T_1_ – T_2_)	−0.163	0.014	−11.602	<0.001*			Exercise (T_0_ − T_1_)	0.101	0.004	23.555	<0.001*		
Exercise condition × exercise	0.000	0.009	0.015	0.988			Recovery (T_1_ – T_2_)	−0.163	0.006	−25.123	<0.001*		
Exercise condition × recovery	−0.014	0.014	−0.999	0.384			Female IR-male IR × exercise	0.011	0.012	0.929	0.504		
							Female CR-male CR × exercise	0.025	0.012	2.059	0.079		
							Female IR-male IR × recovery	−0.035	0.018	−1.896	0.097		
							Female CR-male CR × recovery	0.001	0.018	0.070	0.944		
NLR_robust_							*NLR*						
(Intercept)	0.278	0.028	10.078	<0.001*	Participant	0.122	(Intercept)	0.190	0.011	17.787	<0.001*	Participant	0.000
Exercise (T_0_ − T_1_)	−0.041	0.023	−1.770	0.092	Residual	0.110	Baseline	0.863	0.058	14.792	<0.001*	Residual	0.092
Recovery ( T_1_ – T_2_)	0.572	0.035	16.411	<0.001*			Exercise (T_0_ − T_1_)	−0.037	0.019	−1.947	0.086		
Exercise condition × exercise	0.026	0.023	1.119	0.263			Recovery (T_1_ – T_2_)	0.600	0.029	20.768	<0.001*		
Exercise condition × recovery	−0.159	0.035	−4.570	<0.001*			Female IR-male IR × exercise	−0.059	0.054	−1.084	0.333		
							Female CR-male CR × exercise	−0.056	0.054	−1.037	0.333		
							Female IR-male IR × recovery	−0.032	0.081	−0.400	0.689		
							Female CR-male CR × recovery	−0.251	0.082	−3.053	0.005*		
PLR							PLR_robust_						
(Intercept)	2.177	0.029	75.919	<0.001*	Participant	0.136	(Intercept)	0.118	0.076	1.562	0.296	Participant	0.000
Exercise (T_0_ − T_1_)	−0.045	0.017	−2.741	0.010*	Residual	0.081	Baseline	0.973	0.036	27.272	<0.001*	Residual	0.064
Recovery (T_1_ – T_2_)	0.267	0.025	10.673	<0.001*			Exercise (T_0_ − T_1_)	−0.038	0.013	−2.875	0.013*		
Exercise condition × exercise	0.012	0.017	0.720	0.473			Recovery (T_1_ – T_2_)	0.261	0.020	12.939	<0.001*		
Exercise condition × recovery	−0.073	0.025	−2.937	0.008*			Female IR-male IR × exercise	−0.012	0.038	−0.319	0.750		
							Female CR-male CR × exercise	0.015	0.038	0.401	0.750		
							Female IR-male IR × recovery	−0.040	0.057	−0.703	0.603		
							Female CR-male CR × recovery	−0.069	0.057	−1.204	0.381		
SII_robust_							*SII*						
(Intercept)	2.653	0.035	75.082	<0.001*	Participant	0.153	(Intercept)	0.433	0.111	3.914	<0.001*	Participant	0.000
Exercise (T_0_ − T_1_)	0.065	0.024	2.729	0.008*	Residual	0.135	Baseline	0.900	0.045	20.142	<0.001*	Residual	0.094
Recovery (T_1_ – T_2_)	0.420	0.036	11.656	<0.001*			Exercise (T_0_ − T_1_)	0.059	0.020	2.995	0.005*		
Exercise condition × exercise	0.031	0.024	1.280	0.201			Recovery (T_1_ – T_2_)	0.441	0.030	14.830	<0.001*		
Exercise condition × recovery	−0.172	0.036	−4.773	<0.001*			Female IR-male IR × exercise	−0.055	0.056	−0.981	0.408		
							Female CR-male CR × exercise	−0.003	0.056	−0.047	0.962		
							Female IR-male IR × recovery	−0.063	0.084	−0.752	0.502		
							Female CR-male CR × recovery	−0.257	0.085	−3.038	0.005*		
Cortisol							Cortisol_robust_						
(Intercept)	1.997	0.031	64.398	<0.001*	Participant	0.147	(Intercept)	0.263	0.095	2.758	0.029*	Participant	0.000
Exercise (T_0_ − T_1_)	−0.004	0.018	−0.212	0.832	Residual	0.090	Baseline	0.857	0.047	18.333	<0.001*	Residual	0.090
Recovery (T_1_ – T_2_)	−0.083	0.028	−2.972	0.005*			Exercise (T_0_ − T_1_)	−0.005	0.019	−0.250	0.892		
Exercise condition × exercise	−0.061	0.018	−3.331	0.002*			Recovery (T_1_ – T_2_)	−0.070	0.028	−2.452	0.047*		
Exercise condition × recovery	0.006	0.028	0.212	0.832			Female IR-male IR × exercise	0.034	0.053	0.639	0.886		
							Female CR-male CR × exercise	0.005	0.053	0.096	0.923		
							Female IR-male IR × recovery	0.047	0.080	0.592	0.886		
							Female CR-male CR × recovery	0.024	0.081	0.302	0.892		

Complete statistical computations are based on log10 transformed values. Contrasts exercise (T_1_) and recovery (T_2_) reflect whether mean values at the time-point differ from the mean of the previous time-point (T_0_ − T_1_/T_1_ − T_2_). Exercise condition × time-point contrasts reflect whether these time effects differ between conditions. Female IR/female CR—male IR/male CR × time-point contrasts reflect whether the means in the female groups differ to the means of the males in the corresponding condition. Under statistic t-values are reported. Robust analyses are indicated by robust behind the parameters name

*IR* interval running, *CR* continuous running, *sd* standard deviation, *se* standard error

* indicates a *p*-value < 0.05 (in bold)

**Table 4:** Post hoc comparisons of time and between exercise condition and sex × condition effects

Variable	Contrast			Statistic	df	*p*			
Within exercise condition effects									
Leukocytes	1 h post	–	Pre	5.590	114.094	<0.001*			
Lymphocytes	1 h post	–	Pre	−8.726	114.054	<0.001*			
Neutrophils	1 h post	–	Pre	10.256	114.131	<0.001*			
Platelets	1 h post	–	Pre	−1.042	114.015	0.300			
NLR _robust_	1 h post	–	Pre	14.652	inf	<0.001*			
PLR	1 h post	–	Pre	7.949	114.032	<0.001*			
SII _robust_	1 h post	–	Pre	14.400	inf	<0.001*			
Cortisol	1 h post	–	Pre	−3.183	114.034	0.004*			

Variable	Contrast			Post			1 h Post		
				Statistic	df	*p*	Statistic	df	*p*
Between exercise condition									
Lymphocytes	IR	–	CR	1.554	114.000	0.123	−1.883	114.106	0.062
Neutrophils	IR	–	CR	0.022	114.002	0.982	3.854	114.258	0.000*
NLR _robust_	IR	–	CR	−0.795	Inf	0.426	5.637	Inf	<0.001*
PLR	IR	–	CR	−1.558	114.000	0.122	2.589	114.064	0.011*
SII _robust_	IR	–	CR	−0.917	Inf	0.359	5.802	Inf	<0.001*
Cortisol	IR	–	CR	5.088	114.000	<0.001*	5.088	114.000	<0.001*
Between sex × condition									
Neutrophils _robust_	Female IR	–	male IR	−0.737	Inf	0.461	−4.021	Inf	<0.001*
	Female CR	–	male CR	−0.740	Inf	0.461	−5.998	Inf	<0.001*
NLR _robust_	Female CR	–	male CR	−1.084	Inf	0.316	−5.257	Inf	<0.001*
SII _robust_	Female CR	–	male CR	−0.071	Inf	0.943	−4.319	Inf	<0.001*

Complete statistical computations are based on log10 transformed values. For robust *t*-tests (indicated by robust behind the parameters name) the z-ratio instead of *t*-values is reported under statistic

*IR* interval running, *CR* continuous running, *df* degrees of freedom

* indicates a *p*-value < 0.05 (in bold)

LYM counts increased after exercise in both conditions and decreased below baseline until 1 h post (Fig. 2b). The observed interaction effect was not confirmed by post hoc analysis (Tables 3, 4). There was also no statistical difference between the sexes (Fig. 2f; Table 3).

NEUT counts increased over all time points in both exercise conditions when compared to pre values, with significantly higher values for IR compared to CR 1 h post exercise (Fig. 2c; Tables 3, 4). Regarding sex-dependent differences, NEUT counts were significantly higher in females after IR compared to males after IR 1 h post-exercise cessation. This difference was also evident after CR, where males had significantly lower levels than females 1 h post exercise (Fig. 2g; Tables 3, 4).

PLT counts were observed to increase from pre to post exercise, but returned to baseline until 1 h post (Fig. 2d; Table 3). The same kinetic is observable in conditions separated by sex (Fig. 2h; Table 3). Statistically significant differences were not observed between conditions or between sexes (Table 3).

**Fig. 3:**
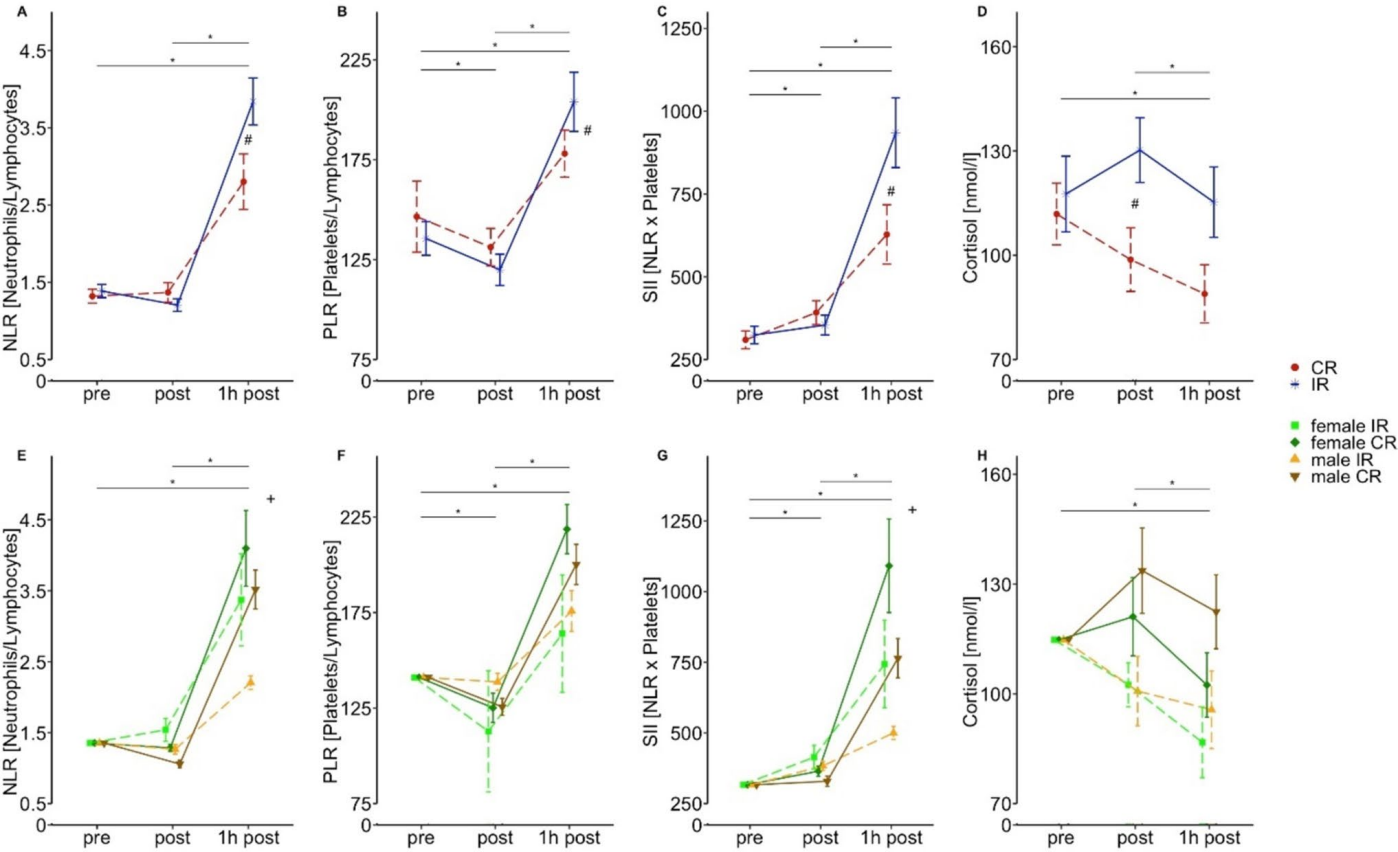
Effects of exercise condition and sex-dependent differences in the kinetic of the cellular immune inflammation markers and cortisol level. Data are presented as means ± SE. * indicates significant main effects of time (*p* < 0.05). **#** indicates significant time × condition interaction effects (*p* < 0.05). Significance codes for sex-dependent interactions (*p* < 0.05): Ω = female IR vs male IR, + = female CR vs male CR

Looking at the NLR, 1 h post exercise both conditions increased with significantly higher values after IR compared to CR (Fig. 3a; Tables 3, 4). Regarding the sex-dependent effects the responses of females and males upon IR were similar. In contrast, 1 h post-exercise cessation the male CR group had significantly lower values compared to the female CR group (Fig. 3e; Tables 3, 4).

A decrease of PLR was observed immediately after exercise cessation with increasing values 1 h post exercise (Fig. 3b). A significant difference between conditions was present 1 h post exercise, demonstrating higher values after IR compared to CR (Fig. 3b; Tables 3, 4). However, no statistical differences were observed between females and males after IR or CR, following the same kinetics described above (Fig. 3f; Table 3).

The SII increased significantly immediately from pre to post, up to 1 h post exercise (Fig. 3c; Table 3). At 1 h post exercise, a significant difference between both exercise conditions was evident, demonstrating even higher values after IR compared to CR (Fig. 3c). However, no difference was observed between females and males who did IR (Table 3). Instead, females performing CR showed significantly higher values compared to males who did CR 1 h post-exercise cessation (Fig. 3g; Table 3).

Cortisol levels decreased statistically significant 1 h post-exercise cessation (Fig. 3d; Tables 3, 4). A significant difference between the two exercise conditions was detected immediately after exercise with cortisol values significantly higher after IR compared to CR (Tables 3, 4). However, the analysis of covariances did not yield any significant sex-dependent differences (Table 3).

## Discussion

In line with our hypothesis, IR provokes stronger perturbations in circulating immune cells (especially NEUT) compared to CR when mean intensity and duration was matched. In general, the exercise-induced immune cell kinetic corresponds to the current literature of exercise induced alterations, since (1) an increase in LEUK counts is observable immediately after exercise, (2) a sustained increase in NEUT is observable until 1 h after exercise, (3) an increase in LYM counts is followed by a decrease in LYM counts immediately after exercise (Gabriel et al. 1992; Malm et al. 2000; Nieman et al. 1995; Shek et al. 1995; Walzik et al. 2021). Despite this well-described immunological response to acute aerobic exercise, our results provide additional evidence that, beyond the training modality, biological sex also has an influence on transient alterations in immune cells. Our results indicate that specifically NEUT responded stronger in females independent of the running protocol (IR, CR).

However, our findings differ from those of Bogdanis et al. (2022), who firstly also matched the mean intensity and duration when investigating circulating immune cells. The intervention of Bogdanis et al. (2022) varies from ours, which could explain the differences in the results. While we compared three-minute interval bouts at 90% ${\dot{\text{V}}}$O_2_peak with a 50-min CR at 70% ${\dot{\text{V}}}$˙O_2_peak (matched for 50 min and a mean intensity of 70% ${\dot{\text{V}}}$˙O_2_peak), Bogdanis et al. (2022) compared shorter interval bouts (60 s, 30 s and 10 s) at 100% ${\dot{\text{V}}}$˙O_2_max with a 26-min CR at a lower intensity of 49% ${\dot{\text{V}}}$O_2_max (matched for 26 min and a mean intensity of 49%). They observed a significantly higher increase of LEUK counts immediately after exercise only in intervals with 60 s bouts, while values decreased back to baseline in all conditions. Similar, LYM counts significantly increased after 60 s interval bouts compared to the other conditions, since 10 s interval bouts and continuous exercise did not even induce any alterations. Compared to our findings, differences of interval and continuous running in LEUK and LYM counts dissolve, suggesting both of our regimes to be potent stressors. This seems to be especially true for PLT counts, as we observed an increase post exercise without differences between conditions, while Bogdanis et al. (2022) did not observed any significant increase immediately after exercise. Most interestingly, we observed a stronger mobilization of NEUT counts by IR compared to CR despite matching both interventions in terms of duration and mean intensity. In contrast, exercise regimes applied by Bogdanis et al. (2022) did not show any differences and NEUT counts decreased already after 1 h post exercise. In summary, the stronger perturbations in circulating immune cells observed in our study can be explained by the overall higher intensity, longer duration of intervals, and therewith higher overall workload. In contrast to our exercise modality, Bogdanis et al. (2022) conducted their intervention on a cycling ergometer involving less active muscle mass.

Potential underlying mechanisms for the more pronounced immune cell mobilization of NEUT induced by IR could be driven by stress response hormones like glucocorticoids and catecholamines as well as mechanical shear stress. Intensity dependent increases in stress hormones are already well established (Walsh et al. 2011a). A higher secretion in response to the higher peak intensities elicit by IR may mobilize NEUT from marginal pools into the bloodstream (Peake et al. 2017; Simpson et al. 2015). However, while we observed a significant difference of cortisol levels between IR and CR, no increase from pre to any point after exercise was observed in the present study. In detail, cortisol levels after CR were decreasing compared to pre-exercise levels as well as compared to the IR condition. This suggests that cortisol secretion may not be the primary factor of the observed exercise-induced increase in NEUT counts. It should be considered that a detailed kinetic of the cortisol levels during or shortly after exercise cessation could provide further insights into potentially missed trajectories.

In a crossover design study by Peake et al. (2014), a work-matched interval vs. continuous cycling exercise intervention has shown higher stress response hormones, among cortisol and adrenalin, after the interval exercise compared to continuous exercise. Therefore, we suggest to include more stress response hormones as well as the investigation of mechanical stress parameters in future studies as they seem to have a more relevant role.

Regarding sex differences to mean intensity and duration matched exercise regimes, a greater response of females can be seen especially in NEUT counts. In detail, this increase was more pronounced in females compared to males in both exercise regimes. The literature on sex-dependent exercise-induced immune responses is limited, but Timmons et al. (2005) investigated the number of circulating NEUT after 90 min of cycling at 65% ${\dot{\text{V}}}$˙O_2_max and observed a stronger response in females compared to males. The observed sex differences appeared to be due to the greater increase in NEUT counts after exercise in females compared to males, which is in line with our results. It has been described that higher cortisol levels measured when oral contraceptives are taken (Notbohm et al. 2023a). Therefore, we have additionally analyzed the two females who use contraceptives (Online Resource 3). However, removing these two women from the overall analysis does not change the results (results not shown).

Differences in the sex hormones could be another possible explanation as the immune cells themselves have corresponding receptors. Immune cells are known to be directly controlled by estrogen or testosterone, whereby e.g. estrogen increases the expression of pro-inflammatory cytokines, while testosterone tends to have opposite effects (Klein et al. 2010). Furthermore, positive correlations have been observed for exercise induced alterations in female sex hormones and immune cell counts (B cells, NK cells), particularly after cycling during the late follicular phase of menstruation (Bouillon et al. 2006). The results indicate a higher immune reactivity of females compared to males in response to exercise, which is already known in reaction to pathogens and vaccinations (Cortes and Miguel 2022; Fragala et al. 2011; Klein and Flanagan 2016). Further potential moderators of sex-dependent differences in response to exercise should be included in future investigations. For example, Notbohm et al. reported different responses of various components of the immune system in dependence on the menstrual cycle phase and the use of oral contraceptives (Notbohm et al. 2023a, 2023b). The consideration of sex-dependent differences in the exercise-induced alterations of circulating immune cells enables a more differentiated depiction of individual immunological processes.

Both NLR and SII, in comparison to PLR, are suitable to show the observed exercise effect on NEUT counts. As NEUT represent one of the key nonspecific host defense cell populations with additional influence on T cell and B cell activities in our immune system, our findings underline the importance and suitability of the cellular immune inflammation markers NLR and SII to monitor the immune status following acute exercise sessions of different intensities and in different sexes. This is especially important when dealing with recovery durations to consecutive exercise sessions. To understand the longer term changes in the cellular immune inflammation markers, future studies should also test for changes over 24 h and even longer, as these remain at high levels after the end of exercise.

## Conclusion

Compared to a mean intensity and duration matched CR, the IR protocol induces a greater immune cell mobilization. The immune cell mobilization is primarily based on a systemic increase in NEUT counts. This effect is clearly detectable by the cellular immune inflammation markers NLR and SII. In addition, biological sex influences the response of immune cells to acute exercise, with stronger responses in female than males. These findings are of relevance for trainers, exercise physiologists and clinicians who should consider conclusive blood markers as comprehensive tools to give individual exercise recommendations and monitor the immunological training load.

## Supplementary Information

Below is the link to the electronic supplementary material.

Supplementary file1 (DOCX 18 KB)

Supplementary file2 (DOCX 26 KB)

Supplementary file3 (JPEG 155 KB)

## Data Availability

The data, code book, and analytic code that support the findings of this study are available on request from the corresponding author.

## Abbreviations

BMI: Body mass index
CR: Continuous running
FDR: False discovery rate
IR: Interval running
LEUK: Leukocyte
LYM: Lymphocyte
NEUT: Neutrophil
NLR: Neutrophil-to-lymphocyte ratio
PLR: Platelets-to-lymphocyte ratio
SD: Standard deviation
SE: Standard error
SII: Systemic immune-inflammation index
${\dot{\text{V}}}$O_2_max: Maximal oxygen uptake
${\dot{\text{V}}}$˙O_2_peak: Peak oxygen uptake
