# Supplementary material for: Neutrophil but not lymphocyte response to matched interval and continuous running differs between protocols and sex
Source: Eur J Appl Physiol. 2024 Dec 3;125(5):1271–82. doi: 10.1007/s00421-024-05675-0 (PMC12055875; doi:10.1007/s00421-024-05675-0)
Supplement: Supplementary file 1 — Supplementary file1 (DOCX 18 KB) [file 421_2024_5675_MOESM1_ESM.docx]

**Online Resource 1** Means and standard deviation of variables by condition

|  |  | **CR** | | | | **IR** | | | |
| --- | --- | --- | --- | --- | --- | --- | --- | --- | --- |
| **variable** | **timepoint** | **n** | **mean** | **sd** | **n** | | **mean** | **sd** |  |
| Leukocytes | pre | 24 | 4.48 | 0.84 | 24 | | 4.75 | 1.10 |  |
| Leukocytes | post | 24 | 5.96 | 1.05 | 24 | | 6.16 | 1.42 |  |
| Leukocytes | 1h post | 23 | 5.29 | 1.55 | 24 | | 6.24 | 1.84 |  |
| Lymphocytes | pre | 24 | 1.77 | 0.46 | 24 | | 1.82 | 0.52 |  |
| Lymphocytes | post | 24 | 2.45 | 0.84 | 24 | | 2.62 | 0.79 |  |
| Lymphocytes | 1h post | 23 | 1.35 | 0.37 | 24 | | 1.24 | 0.40 |  |
| Neutrpohils | pre | 24 | 2.19 | 0.56 | 24 | | 2.42 | 0.72 |  |
| Neutrpohils | post | 24 | 2.96 | 0.74 | 24 | | 2.97 | 0.77 |  |
| Neutrpohils | 1h post | 23 | 3.47 | 1.41 | 24 | | 4.50 | 1.61 |  |
| Platelets | pre | 24 | 235.29 | 62.48 | 24 | | 235.83 | 68.02 |  |
| Platelets | post | 24 | 296.08 | 81.39 | 24 | | 297.63 | 93.52 |  |
| Platelets | 1h post | 23 | 227.65 | 54.70 | 24 | | 238.04 | 78.05 |  |
| NLR | pre | 24 | 1.32 | 0.44 | 24 | | 1.39 | 0.42 |  |
| NLR | post | 24 | 1.37 | 0.62 | 24 | | 1.21 | 0.40 |  |
| NLR | 1h post | 23 | 2.80 | 1.72 | 24 | | 3.84 | 1.49 |  |
| PLR | pre | 24 | 146.55 | 87.10 | 24 | | 135.60 | 41.21 |  |
| PLR | post | 24 | 131.26 | 45.99 | 24 | | 119.93 | 38.44 |  |
| PLR | 1h post | 23 | 178.08 | 56.36 | 24 | | 203.97 | 72.54 |  |
| SII | pre | 24 | 309.73 | 131.60 | 24 | | 324.18 | 129.32 |  |
| SII | post | 24 | 391.76 | 176.17 | 24 | | 354.36 | 146.77 |  |
| SII | 1h post | 23 | 628.03 | 428.81 | 24 | | 935.17 | 516.00 |  |
| Cortisol | pre | 24 | 111.88 | 43.52 | 24 | | 117.63 | 53.28 |  |
| Cortisol | post | 24 | 98.75 | 44.84 | 24 | | 130.25 | 45.60 |  |
| Cortisol | 1h post | 23 | 88.91 | 40.09 | 24 | | 115.29 | 49.55 |  |

Abbreviations: IR: interval running, CR: continuous running, n: number of cases, sd: standard deviation.
