# Supplementary material for: Neutrophil but not lymphocyte response to matched interval and continuous running differs between protocols and sex
Source: Eur J Appl Physiol. 2024 Dec 3;125(5):1271–82. doi: 10.1007/s00421-024-05675-0 (PMC12055875; doi:10.1007/s00421-024-05675-0)
Supplement: Supplementary file 2 — Supplementary file2 (DOCX 26 KB) [file 421_2024_5675_MOESM2_ESM.docx]

**Online Resource 2** Means and standard deviation of variables by condition and sex

|  |  | **female** | | |  | **male** | | |
| --- | --- | --- | --- | --- | --- | --- | --- | --- |
| **variable** | **timepoint** | **n** | **mean** | **sd** |  | **n** | **mean** | **sd** |
| **interval running** |  |  |  |  |  |  |  |  |
| Leukocytes | pre | 12 | 4.65 | 1.12 |  | 12 | 4.86 | 1.12 |
| Leukocytes | post | 12 | 6.03 | 1.28 |  | 12 | 6.30 | 1.60 |
| Leukocytes | 1h post | 12 | 6.65 | 2.04 |  | 12 | 5.83 | 1.61 |
| Lymphocytes | pre | 12 | 1.92 | 0.48 |  | 12 | 1.71 | 0.55 |
| Lymphocytes | post | 12 | 2.70 | 0.76 |  | 12 | 2.55 | 0.85 |
| Lymphocytes | 1h post | 12 | 1.35 | 0.51 |  | 12 | 1.13 | 0.22 |
| Neutrophiles | pre | 12 | 2.19 | 0.67 |  | 12 | 2.64 | 0.72 |
| Neutrophiles | post | 12 | 2.80 | 0.74 |  | 12 | 3.13 | 0.79 |
| Neutrophiles | 1h post | 12 | 4.81 | 1.79 |  | 12 | 4.20 | 1.42 |
| Platelets | pre | 12 | 260.17 | 76.02 |  | 12 | 211.50 | 51.01 |
| Platelets | post | 12 | 327.00 | 107.75 |  | 12 | 268.25 | 69.23 |
| Platelets | 1h post | 12 | 270.25 | 88.46 |  | 12 | 205.83 | 51.48 |
| NLR | pre | 12 | 1.16 | 0.32 |  | 12 | 1.61 | 0.39 |
| NLR | post | 12 | 1.10 | 0.39 |  | 12 | 1.31 | 0.39 |
| NLR | 1h post | 12 | 3.91 | 1.81 |  | 12 | 3.77 | 1.17 |
| PLR | pre | 12 | 140.53 | 46.38 |  | 12 | 130.68 | 36.71 |
| PLR | post | 12 | 124.60 | 36.13 |  | 12 | 115.25 | 41.67 |
| PLR | 1h post | 12 | 218.13 | 83.49 |  | 12 | 189.82 | 59.94 |
| SII | pre | 12 | 301.05 | 114.31 |  | 12 | 347.31 | 143.99 |
| SII | post | 12 | 349.05 | 142.03 |  | 12 | 359.68 | 157.50 |
| SII | 1h post | 12 | 1075.55 | 623.12 |  | 12 | 794.79 | 354.17 |
| Cortisol | pre | 12 | 116.42 | 67.76 |  | 12 | 118.83 | 36.61 |
| Cortisol | post | 12 | 122.75 | 54.02 |  | 12 | 137.75 | 36.15 |
| Cortisol | 1h post | 12 | 104.08 | 60.84 |  | 12 | 126.50 | 34.02 |
| **continuous running** |  |  |  |  |  |  |  |  |
| Leukocytes | pre | 12 | 4.50 | 1.09 |  | 12 | 4.47 | 0.52 |
| Leukocytes | post | 12 | 6.07 | 1.17 |  | 12 | 5.86 | 0.95 |
| Leukocytes | 1h post | 12 | 5.68 | 1.98 |  | 11 | 4.86 | 0.78 |
| Lymphocytes | pre | 12 | 1.89 | 0.56 |  | 12 | 1.66 | 0.31 |
| Lymphocytes | post | 12 | 2.49 | 0.83 |  | 12 | 2.40 | 0.88 |
| Lymphocytes | 1h post | 12 | 1.38 | 0.45 |  | 11 | 1.32 | 0.27 |
| Neutrophiles | pre | 12 | 2.07 | 0.69 |  | 12 | 2.31 | 0.38 |
| Neutrophiles | post | 12 | 3.01 | 1.01 |  | 12 | 2.91 | 0.33 |
| Neutrophiles | 1h post | 12 | 3.85 | 1.86 |  | 11 | 3.06 | 0.48 |
| Platelets | pre | 12 | 264.58 | 63.94 |  | 12 | 206.00 | 46.91 |
| Platelets | post | 12 | 320.67 | 94.21 |  | 12 | 271.50 | 60.46 |
| Platelets | 1h post | 12 | 241.92 | 61.53 |  | 11 | 212.09 | 43.69 |
| NLR | pre | 12 | 1.20 | 0.48 |  | 12 | 1.44 | 0.38 |
| NLR | post | 12 | 1.38 | 0.76 |  | 12 | 1.36 | 0.47 |
| NLR | 1h post | 12 | 3.22 | 2.34 |  | 11 | 2.35 | 0.29 |
| PLR | pre | 12 | 166.17 | 118.59 |  | 12 | 126.92 | 30.94 |
| PLR | post | 12 | 137.97 | 52.41 |  | 12 | 124.55 | 39.71 |
| PLR | 1h post | 12 | 189.09 | 66.64 |  | 11 | 166.07 | 42.41 |
| SII | pre | 12 | 323.24 | 166.33 |  | 12 | 296.23 | 90.26 |
| SII | post | 12 | 420.63 | 217.99 |  | 12 | 362.90 | 124.73 |
| SII | 1h post | 12 | 750.25 | 571.21 |  | 11 | 494.70 | 90.19 |
| Cortisol | pre | 12 | 115.25 | 57.37 |  | 12 | 108.50 | 25.39 |
| Cortisol | post | 12 | 103.00 | 57.24 |  | 12 | 94.50 | 29.78 |
| Cortisol | 1h post | 12 | 87.25 | 45.12 |  | 11 | 90.73 | 35.92 |

Abbreviations: n: number of cases. sd: standard deviation
