# Supplementary figures and images for: Neutrophil but not lymphocyte response to matched interval and continuous running differs between protocols and sex
Source: Eur J Appl Physiol. 2024 Dec 3;125(5):1271–82. doi: 10.1007/s00421-024-05675-0 (PMC12055875; doi:10.1007/s00421-024-05675-0)

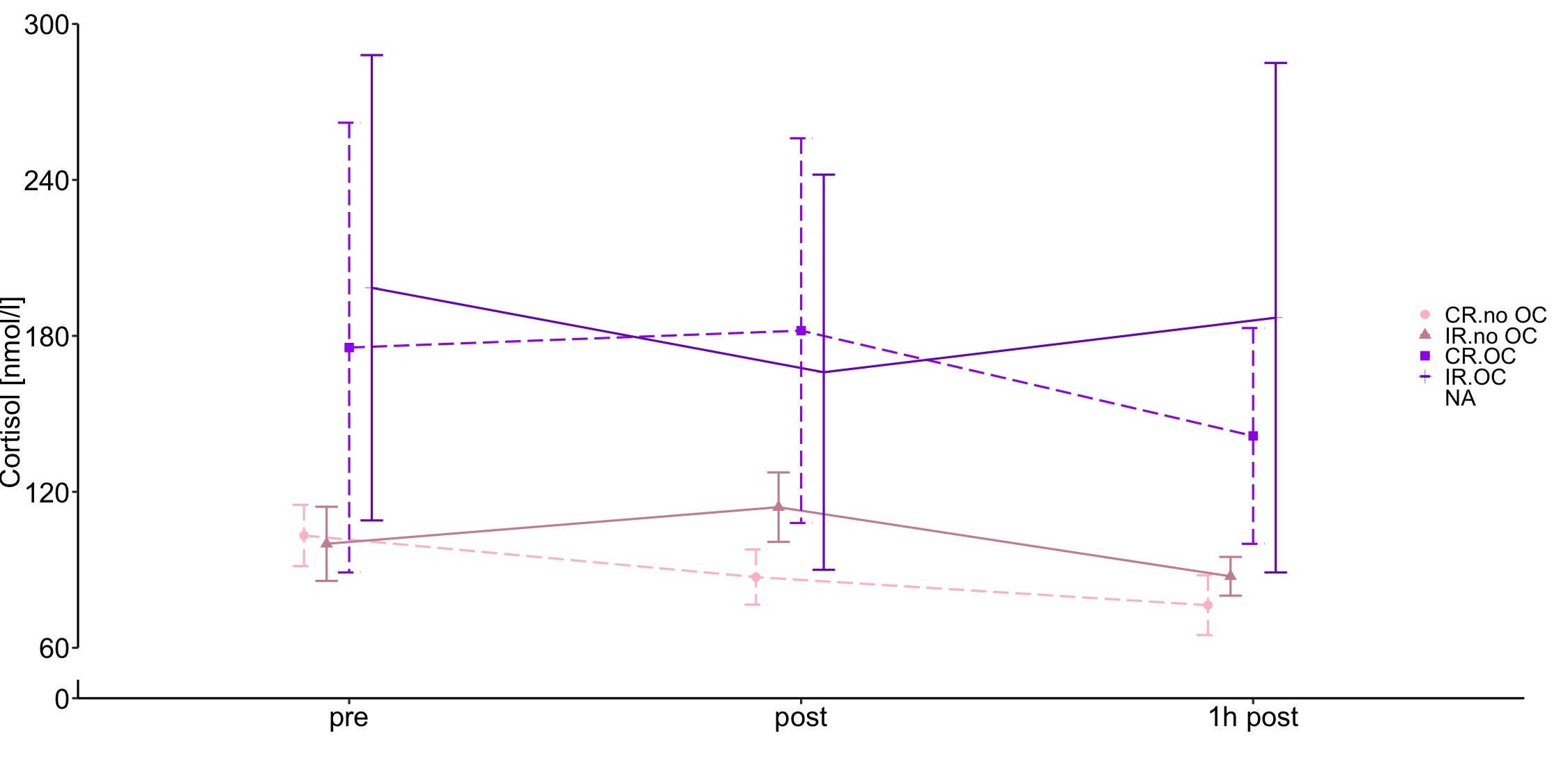

Supplement: Supplementary file 3 — Supplementary file3 (JPEG 155 KB) [file 421_2024_5675_MOESM3_ESM.jpeg]
